# Identification of Cadmium Compounds in a Solution Using Graphene-Based Sensor Array

**DOI:** 10.3390/s23031519

**Published:** 2023-01-30

**Authors:** Tomoya Yoshii, Fuka Nishitsugu, Kazuki Kikawada, Kenzo Maehashi, Takashi Ikuta

**Affiliations:** Institute of Engineering, Tokyo University of Agriculture and Technology, 2-24-16, Nakacho, Koganei, Tokyo 184-8588, Japan

**Keywords:** graphene, cadmium compound, sensor array, heavy metal, terpyridine

## Abstract

Rapid detection of heavy metals in solution is necessary to ensure human health and environmental protection. Some heavy-metal compounds are present in solution as compounds instead of as ions owing to their low ionization. Therefore, the development of sensor devices for the detection of heavy-metal compounds is important. In this study, as a proof of concept, we propose a sensor device using graphene and a chelating agent, which were used to develop an identification technique for three types of cadmium compounds. Pristine-graphene and two types of chelator-modified graphene-based sensors were successfully used to detect cadmium compounds at concentrations ranging from 50 to 1000 μM. The detection time was less than 5 min. The three type of graphene-based sensors responded differently to each cadmium compound, which indicates that they detected cadmium as a cadmium compound instead of as cadmium ions. Furthermore, we successfully identified cadmium compounds by operating these three types of sensors as a sensor array on the same substrate. The results indicate that sensors that focus on heavy-metal compounds instead of heavy-metal ions can be used for the detection of heavy metals in solution.

## 1. Introduction

Heavy metals are highly biotoxic and extremely dangerous, not only to humans but also to all living organisms; therefore, early detection of heavy metals is important [1,2,3]. When a heavy-metal leak occurs, the surrounding environment must be immediately measured. In the case of leakage into water, heavy metals can easily enter living organisms and cause significant damage to humans and environmental systems through heavy-metal poisoning and bioaccumulation. Conventional heavy-metal detection methods, such as X-ray fluorescence analysis and atomic absorption spectrometry, are highly sensitive; however, they are unsuitable for the early detection of heavy metals leaked into water owing to the complex processes involved in their analysis [4,5,6]. The identification of heavy-metal compounds is also required because the toxicity of some heavy metals varies with different compounds. Among heavy metals, cadmium compounds are particularly soluble in water and easily bioaccumulate, which causes various types of pollution, including the Itai-itai disease. The acute toxicity of cadmium exposure can cause critical symptoms even at doses below 10 mg [7]. In addition, the biotoxicity of cadmium compounds differs depending on the type of compound [1,8]. Cadmium compounds are present in solution as compounds instead of as ions owing to their low ionization [9,10,11,12,13]. Therefore, the identification of cadmium compounds in water is important. Conventional methods can determine metal atoms; however, identifying the type of metal compound is difficult, which poses a significant limitation in determining the leakage of heavy metals in water. In addition, sensors focusing on heavy-metal compounds have not been realized in the heavy-metal sensors that were reported in recent years [6,14]. Therefore, this study aims to develop a compact sensor using graphene and chelating agents that is capable of the early detection of cadmium and the identification of cadmium compounds in water.

Graphene is a two-dimensional material composed of carbon atoms, and it has a high potential for sensor applications owing to its high mobility and surface sensitivity (Figures S1 and S2) [15,16,17]. In addition, graphene devices can be easily integrated into other devices using semiconductor fabrication processes, which simplifies the fabrication of sensor arrays [18,19]. In addition, graphene can be modified with organic molecules on its surface via π-stacking, owing to its large π-conjugated system, which is not found in other inorganic semiconductor materials [20,21,22,23]. By taking advantage of these features, previous studies have presented graphene-based sensors optimized for various target molecules, such as antibodies, acid gases, and organic molecules [24,25,26,27]. In graphene-based sensors, the choice of the modified molecule on graphene is key to providing the ability to capture the target molecule. When the target is a heavy metal, chelating agents or aptamers that specifically capture heavy metals are typically used [28,29,30,31]. Chelating agents are molecules that can capture metal atoms using multiple coordination sites, and various molecules have been reported as chelating agents [32,33,34]. Triethylenetetramine-*N,N,N*′,*N*″,*N*‴,*N*‴-hexaacetic acid (TTHA) and terpyridine were used as chelating agents, as these molecules are less water-soluble and can be modified without introducing defects on graphene via CH-π or π-π interaction.

In this study, sensor arrays with pristine or chelator-modified graphene-based sensors are fabricated to identify cadmium compounds in solution. We determine three cadmium compounds by evaluating the response of the sensor array to the introduction of cadmium compounds.

## 2. Materials and Methods

### 2.1. Fabrication of Graphene-Based Sensor Array

**Figure 1:**
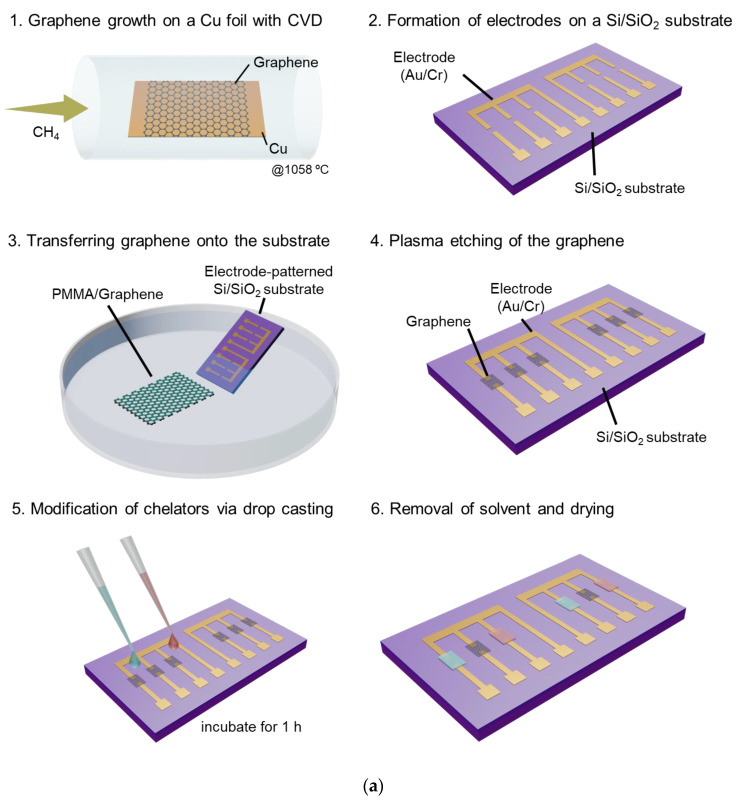

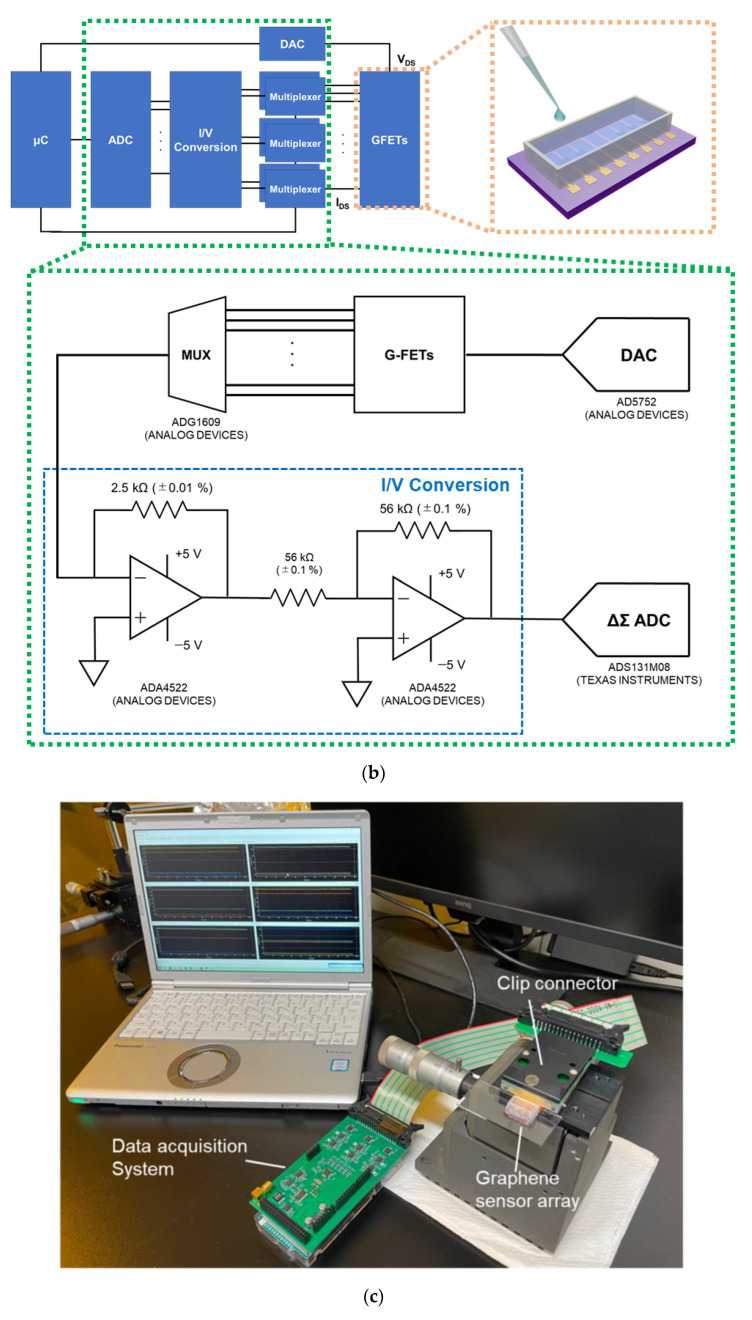
Schematics of (**a**) the fabrication process of the graphene-sensor array, (**b**) the hand-held measurement system, and (**c**) the measurement setup.

Cr/Au electrodes were formed on a Si/SiO_2_ (290 nm) substrate via photolithography and thermal evaporation, as shown in Figure 1a. The photolithography patterns were created using a maskless exposure system (Palet DDB-701-DL, Neoark Corp., Tokyo, Japan). Subsequently, monolayer graphene was synthesized on a copper foil using chemical vapor deposition. The Cu foil was first annealed at 300 °C for 20 min in air. Next, the Cu foils were annealed at 1058 °C for 40 min under 500 sccm of 3% H_2_/97% Ar to remove oxidized impurities and promote catalysis. Graphene was synthesized by flowing 1500 sccm of 3% H_2_/97% Ar with 15 sccm of 5% CH_4_/95% Ar at 1058 °C for 50 min. After the chemical vapor-deposition process, graphene was transferred onto the substrate using a conventional transfer method. Graphene, except for that in the channel area, was removed using an oxygen plasma [26,35]. The channel length and width of the source and drain were both approximately 20 μm. The graphene-sensor array was annealed under Ar/H_2_ at 300 °C for 1 h to remove the residues on the graphene surface, such as the resist.

### 2.2. Modification of Chelating Agents on Graphene

TTHA, and terpyridine were purchased from Tokyo Chemical Industry Co., Ltd., Tokyo, Japan. TTHA was dissolved in 10-mM tris-HCl buffer solution at a concentration of 50 μM, and terpyridine was dissolved in dimethyl sulfoxide at a concentration of 50 μM. The graphene device was annealed again under Ar/H_2_ at 300 °C for 1 h to remove adsorbates. After H_2_ annealing of the graphene-based sensor array, the solution in which these molecules were dissolved was placed onto the graphene via drop casting and modified on the graphene for 1 h. After deposition, the graphene-based sensors array was dried using compressed air and placed in a vacuum chamber overnight to remove residual molecules, such as tris-HCl buffer or dimethyl sulfoxide.

### 2.3. Evaluation of Graphene Device

#### 2.3.1. Electrical Measurement

A graphene-sensor array was placed in a vacuum probe (GRAIL10-VAC-4-LV, Nagase Electronic Equipment Service Co., Ltd. Kanagawa, Japan) for device characterization. The transfer characteristics of the graphene-based sensors were measured using a source-measurement unit (B2912A, Keysight Technologies, Santa Rosa, CA, USA) at 23 °C at 10^−3^ Pa. The field-effect mobility was calculated using the following equation:

(1) $$\mu =\frac{1}{C_{g}}\frac{L}{W}\frac{dI_{DS}}{dV_{BG}},$$

where *C_g_* represents the gate capacitance; *W* and *L* represent the width and length of the channel, respectively; and *I_DS_* and *V_BG_* represent the drain current and back-gate voltage, respectively [36,37,38].

#### 2.3.2. Atomic Force Microscopy and Raman Spectroscopy Analysis

Atomic force microscopy (AFM) and Raman spectroscopy analyses were performed to confirm the modification of the chelating agents on the graphene surface. For pristine graphene, graphene devices were prepared using the above process without modifying the chelating agents, and the AFM (SPM-9700, Shimadzu, Kyoto, Japan) and Raman spectra were measured (NRS-4500, JASCO, Tokyo, Japan). A laser with an excitation wavelength of 523 nm was used for the Raman measurements.

### 2.4. Detection of Cadmium Compound with Graphene-Sensor Array

#### 2.4.1. Handheld Data-Acquisition System

A handheld data-acquisition device was fabricated using a commercially available semiconductor chip. The data-acquisition device consisted of a 6-channel analog-to-digital converter with 24-bit resolution and multiplexer switches, and it is capable of multiple measurements via up to 24 channels. A 16-bit digital-to-analog converter was implemented as a voltage application system. The data-acquisition device was controlled by an Arduino and connected to a clip connector for array operation (Figure 1b).

#### 2.4.2. Measurement of Cadmium Compounds Using Each Graphene-Based Sensors

A pristine graphene and two chelator-modified graphene-based sensors were prepared. The substrate on which the graphene-based sensor was fabricated was fixed to a clip connector and connected to the above data-acquisition system, as shown in Figure 1c. Subsequently, a silicone rubber pool was attached to the substrate, and 200 μL of 100 mM acetic acid buffer solution was added to the silicone rubber pool. 3 M acetate buffer (pH = 5.2) was purchased from Nacalai Tesque, Japan and diluted to 100 mM with ultrapure water. Cadmium chloride and cadmium sulfate were purchased from Fujifilm Wako, Japan, and cadmium nitrate was purchased from Sigma-Aldrich, St. Louis, MO, USA. Each cadmium compound was dissolved in 100 mM acetate buffer, and 20 μL of the solution was dropped into the rubber pool to achieve final concentrations of 10, 50, 100, 500, and 1000 μM. Solutions of each concentration were introduced at 5-min intervals at 23 °C.

#### 2.4.3. Identification of Cadmium Compounds Using Graphene-Based Sensor Array

Three types of graphene-based sensors, pristine, TTHA-modified, and terpyridine-modified, were assembled on a chip with three devices per type on a substrate for identification of cadmium compounds. Nine sensors were operated simultaneously to measure cadmium compounds. The drain voltage was fixed at 50 mV and the sampling interval was 1 s. Each cadmium compound was dissolved in 100 mM acetate buffer, and 20 μL of the solution was dropped into the rubber pool to achieve final concentrations of 10, 50, 100, 500, and 1000 μM at 23 °C. Solutions of each concentration were introduced at 5-min intervals as in Section 2.4.2.

## 3. Results & Discussion

### 3.1. Characterization of Graphene-Based Sensors

**Figure 2:**
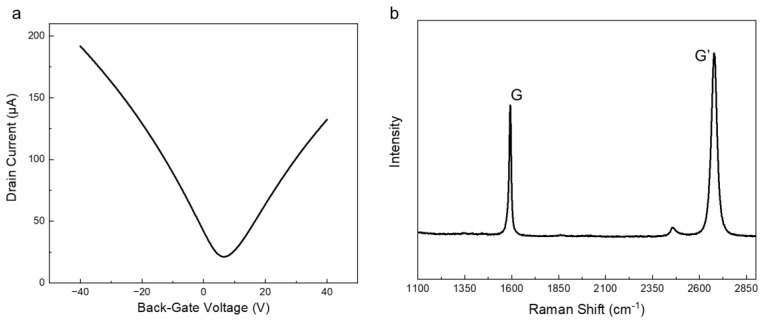
(**a**) Transfer characteristic of the pristine graphene-based sensor. The back-gate voltage changed from −40 V to 40 V under a drain-source voltage of 50 mV. (**b**) A typical Raman spectrum of a pristine graphene on the Si/SiO_2_ substrate.

First, the transfer characteristics of the fabricated graphene-based sensors were measured in a vacuum chamber before they were modified with the chelating agents. The typical transfer characteristics of the pristine graphene-based sensor are shown in Figure 2a. The transfer characteristics exhibit the bipolar characteristics seen in graphene FETs. Moreover, the Dirac point voltage, which is the charge neutral point, was approximately 6 V. Using these results, the field-effect mobilities of holes and electrons were calculated as 7200 and 5800 cm^2^/Vs, respectively. The Raman spectrum of the graphene-based sensor is shown in Figure 2b. The G and G’ peaks are clearly observed, whereas the D peak, which originates from defects in graphene, cannot be observed at 1350 cm^−1^ [39,40]. The full width at half maximum (FWHM) of the G and G’ peaks was calculated to be 13 and 34 cm^−1^, respectively. The intensity ratio of the G and G’ peaks and the FWHM results indicate that the graphene is monolayer graphene [41]. These results reveal that the graphene-based sensor fabricated in this study had few defects and high mobility.

**Figure 3:**
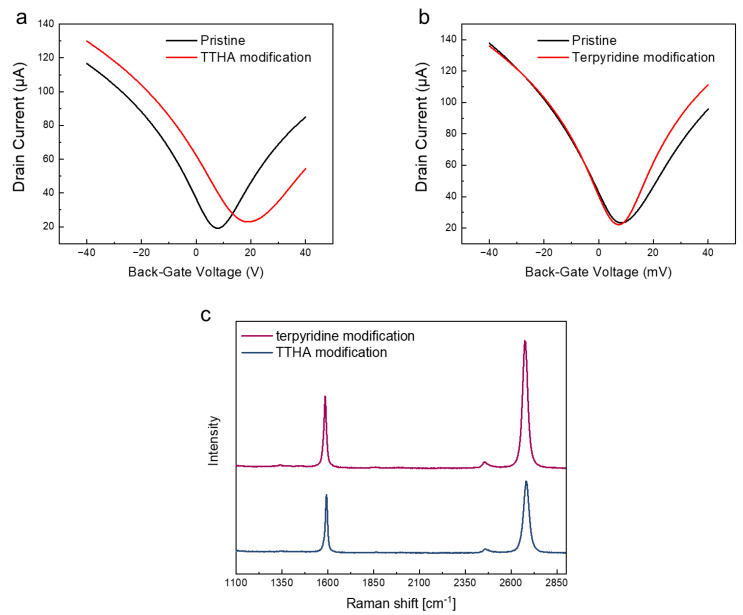
(**a**,**b**) Transfer characteristics of graphene-based sensor before and after TTHA and terpyridine modification, respectively. (**c**) Raman spectra of the TTHA- (indigo) and terpyridine-modified (carmine) graphene-based sensor on a Si/SiO_2_ substrate. Each spectrum is normalized by the G peak intensity.

Figure 3a,b show the transfer characteristics of the graphene-based sensors after chelator modification. In the TTHA-modified graphene-based sensor, the transfer curve shifted in the positive direction (Figure 3a). This is attributed to the hole doping by the oxygen atoms of the carbonyl groups in TTHA. The hole and electron mobilities were approximately 3000 and 2340 cm^2^/Vs, respectively. This decrease in mobility was considered to be owing to increased doping and carrier scattering caused by the modification of TTHA. However, the TTHA-modified graphene retains higher mobility than molecular immobilization via direct chemical modification [42,43,44]. This result suggests that TTHA is immobilized on the graphene owing to CH–π interactions and does not introduce defects into the graphene [19,45]. This is consistent with the Raman spectroscopic results shown in Figure 3c, owing to the absence of a D peak, which indicates that TTHA was successfully modified without introducing defects into graphene. No peaks other than those of graphene were observed in the Raman spectrum after the TTHA modification. This may be owing to the lack of a strong Raman-active mode in TTHA. The terpyridine-modified graphene-based sensor exhibited a few shifts in the transfer characteristics and the hole and electron mobilities of 7050 and 7000 cm^2^/Vs, respectively, as shown in Figure 3b. No significant change in the mobility was observed in the terpyridine-modified device or the TTHA-modified device. A peak was observed in the Raman spectrum obtained after terpyridine modification at approximately 1340 cm^−1^ (Figure 3c) [46,47]. This peak is difficult to distinguish from the D peak of graphene; however, it is considered to be derived from terpyridine because the mobility of the graphene-based sensor is not reduced, and no defects are introduced by the modification via π stacking [25,48].

**Figure 4:**
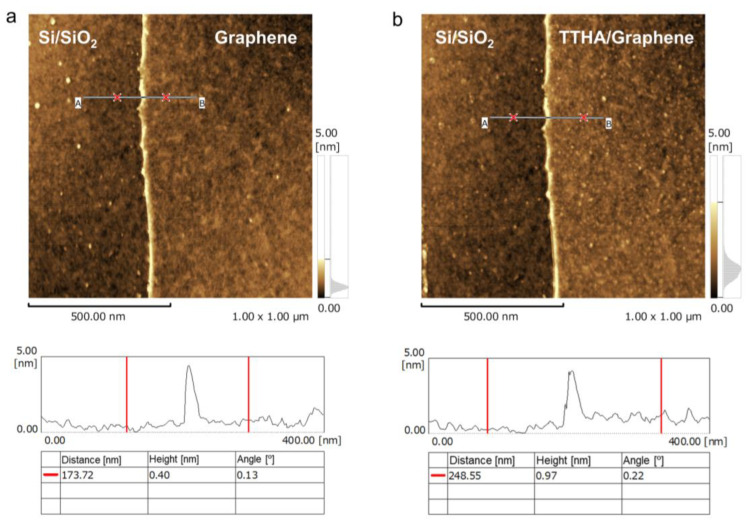

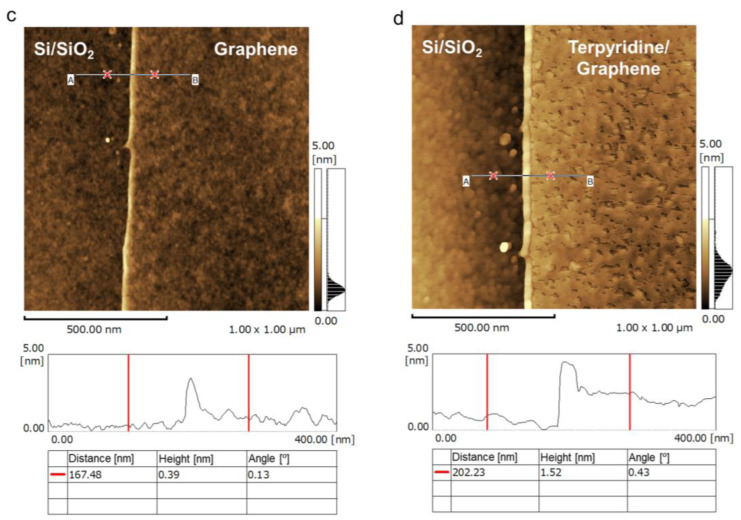
AFM images of (**a**,**c**) pristine graphene and graphene after being modified by (**b**) TTHA and (**d**) terpyridine.

Furthermore, we performed AFM analysis to directly confirm the chelator modification. AFM images before and after THHA and terpyridine modification are shown in Figure 4a–d, respectively. The AFM image of pristine graphene before modification exhibits a thickness of approximately 0.4 nm, which is similar to that of an ideal monolayer of graphene (Figure 4a,c). This is consistent with the Raman-spectroscopy results, which indicate that there was no resist or other residue on the graphene surface. After TTHA modification, the height of the graphene/TTHA film was approximately 1 nm, which was between 2–3 molecular layers thick (Figure 4b). In the case of terpyridine modification, the height of the graphene/terpyridine film was approximately 1.5 nm, which indicates that approximately four or five molecular layers were formed (Figure 4d). Using these results, the root mean square roughness values of the graphene, graphene/TTHA, and graphene/terpyridine films were calculated to be 0.238, 0.321, and 0.178 nm, respectively. These similar values indicate that each molecule was uniformly formed on the graphene without unexpected aggregation. Based on these results, the molecular structures and interaction between the molecules and graphene, the molecules are thought to be modified in parallel with the graphene surface.

### 3.2. Detection of Cadmium Compound with Pristine and Chelating-Agent Modified Graphene

**Figure 5:**
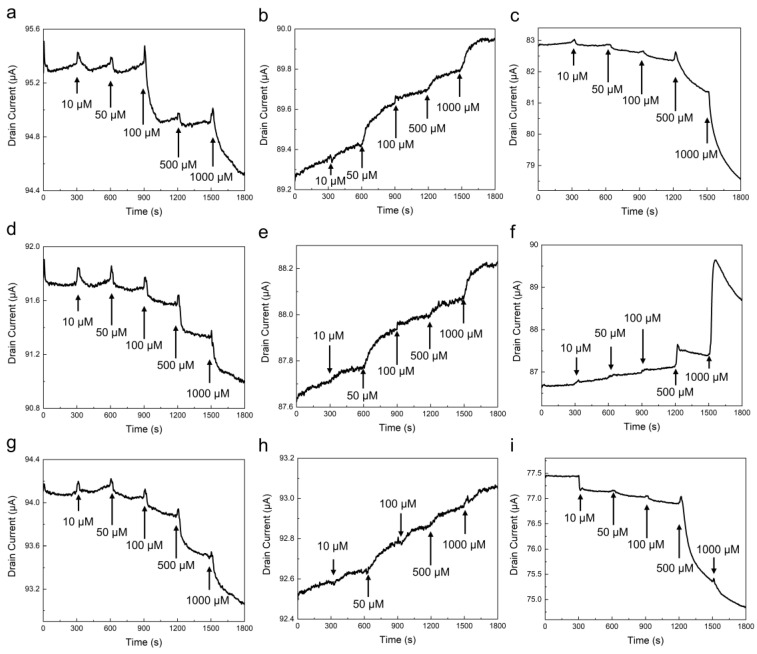
Sensor response of the graphene-based sensors. (**a**–**c**) Pristine graphene with the introduction of cadmium chloride, cadmium nitrate, and cadmium sulfate, respectively. (**d**–**f**) TTHA-modified graphene with the introduction of cadmium chloride, cadmium nitrate, and cadmium sulfate, respectively. (**g**–**i**) Terpyridine-modified graphene with the introduction of cadmium chloride, cadmium nitrate, and cadmium sulfate, respectively.

**Figure 6:**
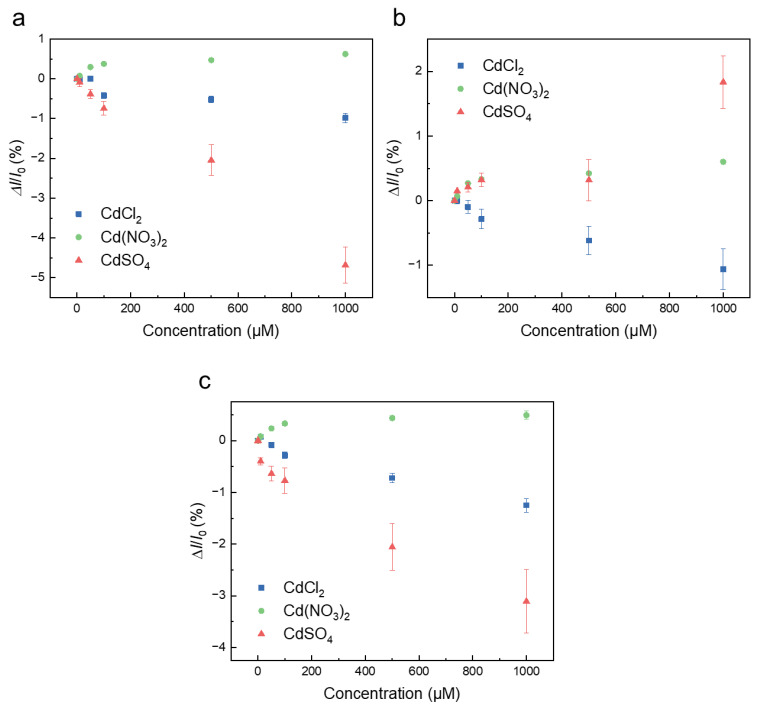
Dependence of the change in drain current on the cadmium concentration in (**a**) pristine, (**b**) TTHA-modified, and (**c**) terpyridine-modified graphene-based sensors.

Graphene-based sensors were used to measure three types of inorganic cadmium salts: cadmium chloride, cadmium sulfate, and cadmium nitrate. A silicon rubber pool was attached to the graphene-based sensor, and measurements were made in acetic acid buffer. A drain voltage of 50 mV was applied, and the time response of the drain current was measured with a sampling interval of 1 s. Figure 5a–c show the measurement results of when cadmium chloride, cadmium sulfate, and cadmium nitrate were introduced to the pristine graphene-based sensors, respectively. No distinct drain-current change was observed in the graphene device with the introduction of 10 μM of any cadmium compound. However, the drain current changed immediately after the introduction of a cadmium compound above 50 μM, and the current almost saturates within 5 min. The drain current of the pristine graphene-based sensor decreased when cadmium chloride and cadmium sulfate were introduced; however, it increased when cadmium nitrate was introduced (Figure 5a–c). Figure 6a–c shows that the drain current of the graphene-based sensor varies with the concentration of each cadmium compound. The drain-current change (ΔI) was normalized to a current value of 0 μM (I_0_) to prevent variations owing to differences in the device characteristics. The results also indicate that the detection limit is between 10–50 μM. Similar to the pristine graphene-based sensor, the drain currents of the TTHA- and terpyridine-modified graphene-based sensors also respond quickly to the introduction of cadmium compounds, as shown in Figure 5d–f. In the TTHA-modified graphene-based sensor, the drain current increased when cadmium nitrate was introduced; however, the drain current decreased for other cadmium compounds (Figure 6b). In the terpyridine-modified graphene-based sensor, the drain current decreased when cadmium chloride and cadmium sulfate were introduced; however, the current increased when cadmium nitrate was introduced, which was similar to the results of the TTHA-modified and pristine graphene-based sensors (Figure 5g–i). The concentration dependence in the terpyridine-modified device is shown in Figure 6c. Similar to the other sensors, the terpyridine-modified graphene-based sensors responded differently to each cadmium compound. These sensor responses were also confirmed using the source measurement units (Figure S3).

**Figure 7:**
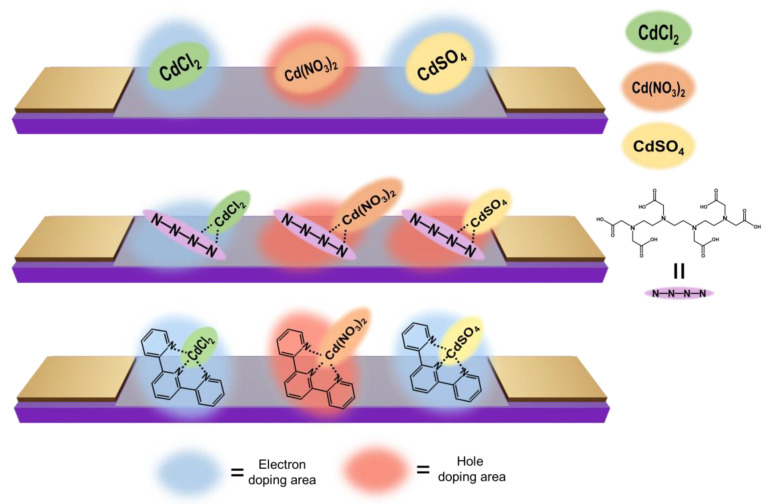
Schematic images of adsorption of cadmium compounds.

The difference in the response of these sensors is may be owing to a change in the electrostatic potential of the chelating agent when the cadmium compound is coordinated to the chelating agent [49,50,51]. When cadmium chloride was adsorbed on the graphene-based sensor, the adsorption was accompanied by electron doping, resulting in a decrease in the drain current. This is considered to be induced by the cationic part of cadmium chloride, as shown in Figure 7. In the case of cadmium nitrate adsorption, holes were induced in the graphene-based sensors owing to the electron-rich nitric acid moiety, resulting in an increase in current. On the other hand, in the case of cadmium sulfate adsorption, electron doping occurs for pristine and terpyridine-modified graphene-based sensors. This is thought to be due to the induction of electrons in the graphene-based sensor by the cationic part of cadmium sulfate, as in the case of cadmium chloride. Conversely, an increase in the drain current is observed for the TTHA-modified graphene-based sensor, indicating that holes are induced by the adsorption of cadmium sulfate on the TTHA-modified graphene-based sensors. The graphene-based sensor reads this change as a transducer and outputs it as a change in drain current [52]. The magnitude of the current change for each sensor was similar for each target. Since the chelating agent is modified on graphene by non-destructive π-π or CH-π interactions rather than by covalent bonding, which is a destructive modification, there is not much difference in the mobility of graphene-based sensors. Therefore, the sensor performance of graphene-based sensors is expected to be similar even when modified with chelating molecules. In addition, these results suggest that the graphene-based sensor detects cadmium as a cadmium compound in solution instead of as a cadmium ion. The responses of our graphene-based sensor to cadmium compounds are summarized in Table 1.

### 3.3. Identification of Cadmium Compound with Graphene-Based Sensor Array

**Figure 8:**
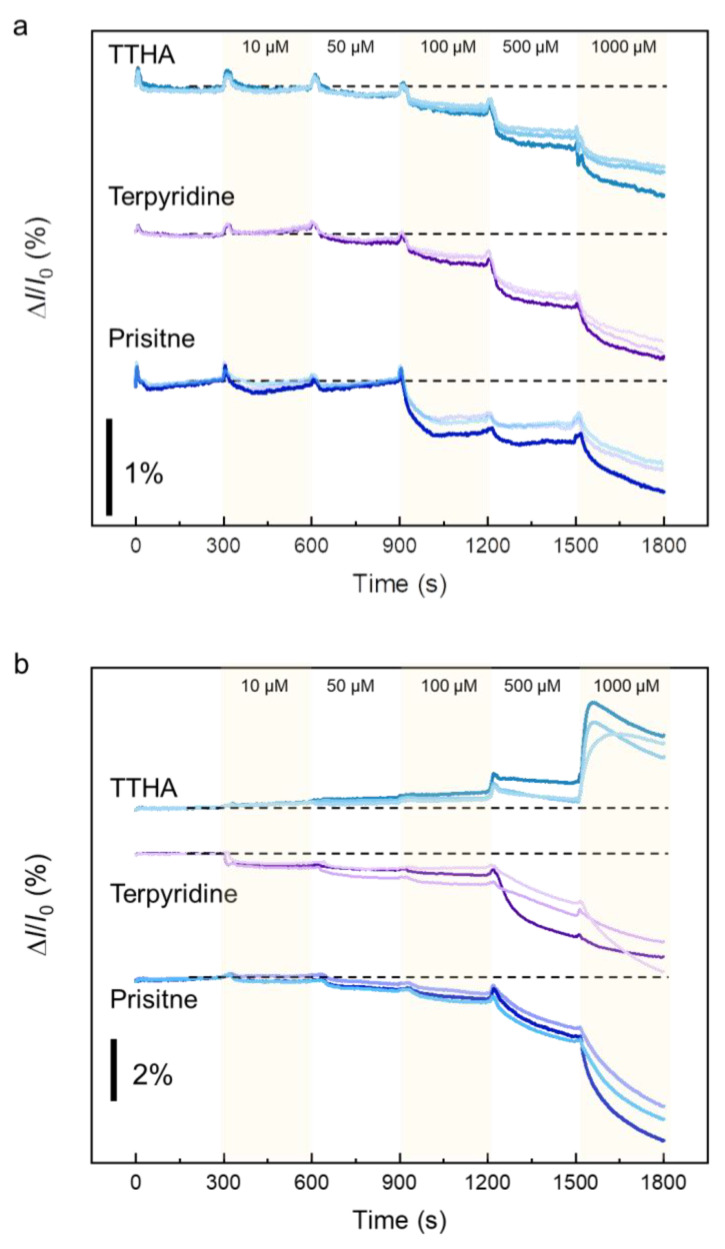

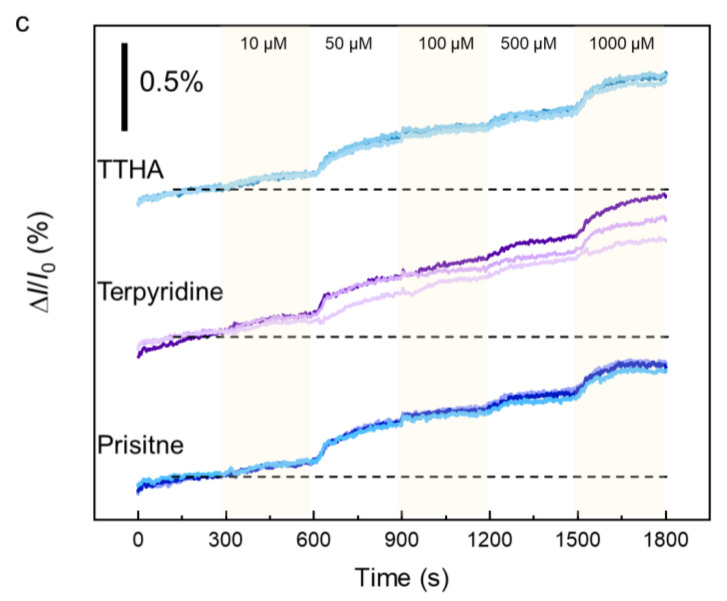
Responses of graphene-sensor arrays to the introduction of (**a**) cadmium chloride, (**b**) cadmium sulfate, and (**c**) cadmium nitrate. The dashed lines represent the initial drain-current values for each.

Based on the above results, the graphene-sensor array was used as a logic circuit to identify cadmium compounds. Three types of graphene-based sensors, pristine, TTHA-modified, and terpyridine-modified, were assembled on one chip with three devices per type on one substrate. We performed simultaneous measurements using nine sensors. These measurements were performed under the same conditions as those in Section 3.2, with a drain voltage of 50 mV and a sampling interval of 1 s. Figure 8a shows the results obtained from the graphene-based sensor arrays after the introduction of cadmium chloride. A decrease in the drain current was observed for all graphene-based sensors. The response of this sensor array and the results given in Table 1 indicate that cadmium chloride can be detected using this sensor array. In the cases of cadmium-sulfate and cadmium-nitrate detection, the sensor-array response was also specific to each cadmium compound (Figure 8b,c). These results indicate that the fabricated graphene-sensor array can be used for the identification of cadmium compounds in solution.

## 4. Conclusions

We fabricated graphene-sensor arrays comprising pristine and chelator-modified graphene for the identification of cadmium compounds in solution, which exhibited different sensor responses to three different cadmium compounds. This indicates that the graphene-based sensors do not detect cadmium as cadmium ions, but as cadmium compounds. This result is consistent with those of previous studies on the state of cadmium compounds in solution. Furthermore, in this study, an array of graphene-based sensors was used to identify cadmium compounds. The sensor arrays exhibited specific responses to each cadmium compound, and we successfully identified the cadmium compounds using the fabricated graphene-sensor arrays. Thus, this study established a detection method for heavy metals that focused on heavy-metal compounds instead of heavy-metal ions. These results represent an important step toward fundamental technologies for the development of sensors for heavy-metal detection. In the future, sensors for other heavy-metal compounds are expected to be realized by optimizing chelating agents.

## Figures and Tables

**Table 1:** Drain-current response of the sensors to each cadmium compound.

Cadmium Compound	Pristine Graphene	TTHA-Modified Grapheme	Terpyridine- Modified Graphene
Cadmium chloride (CdCl_2_)	decrease	decrease	decrease
Cadmium sulfate (CdSO_4_)	decrease	increase	decrease
Cadmium nitrate (Cd(NO_3_)_2_)	increase	increase	increase

## Data Availability

Data of our study are available upon request.

## Supplementary Materials

The following supporting information can be downloaded at: https://www.mdpi.com/article/10.3390/s23031519/s1, Figure S1: Schematic of the detection mechanism in a FET-based sensor; Figure S2: Schematic of the effect of mobility on the drain current change; Figure S3: Transfer characteristics when cadmium chloride was introduced into (a) TTHA-modified and (b) terpyridine-modified graphene. References [21,22] are cited in the supplementary materials.
