# Supplementary material for: Identification of Cadmium Compounds in a Solution Using Graphene-Based Sensor Array
Source: Sensors (Basel). 2023 Jan 30;23(3):1519. doi: 10.3390/s23031519 (PMC9921919; doi:10.3390/s23031519)
Supplement: Supplementary file 1 [file sensors-23-01519-s001.zip › sensors-2177188-supplementary.pdf]

Supplementary Materials

Identification of Cadmium Compounds in a Solution Using Graphene-Based Sensor Array

Tomoya Yoshii, Fuka Nishitsugu, Kazuki Kikawada, Kenzo Maehashi and Takashi Ikuta *

Institute of Engineering, Tokyo University of Agriculture and Technology, 2-24-16, Nakacho, Koganei, Tokyo 184-8588, Japan

* Correspondence: ikuta@go.tuat.ac.jp

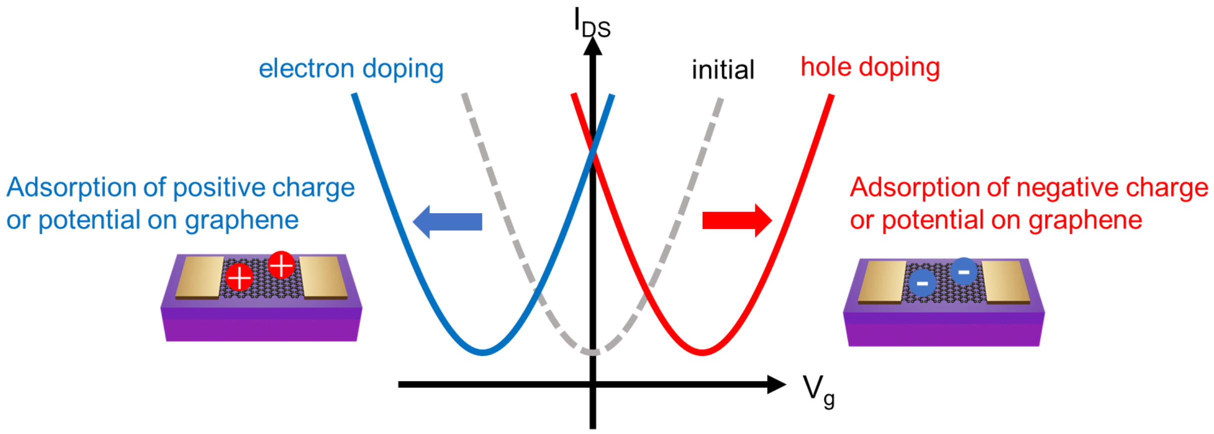

Figure S1. Schematic of the detection mechanism in a FET-based sensor. Transfer characteristics are shifted by positive or negative charge, or electrostatic potential.[1]

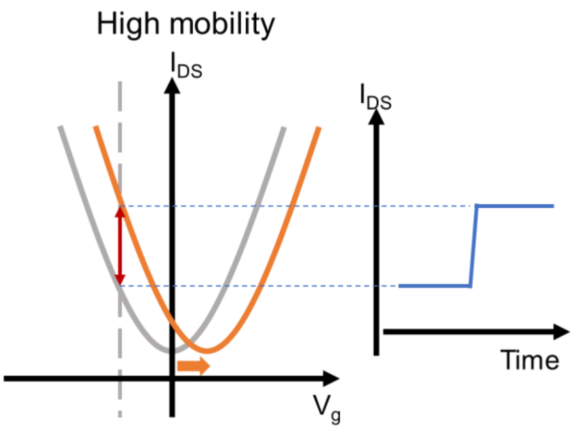

Figure S2. Schematic of the effect of mobility on the drain current change. When the mobility is high, the current change associated with molecular adsorption is large. Therefore, a graphene-based sensor with high mobility is desirable to obtain large signals.

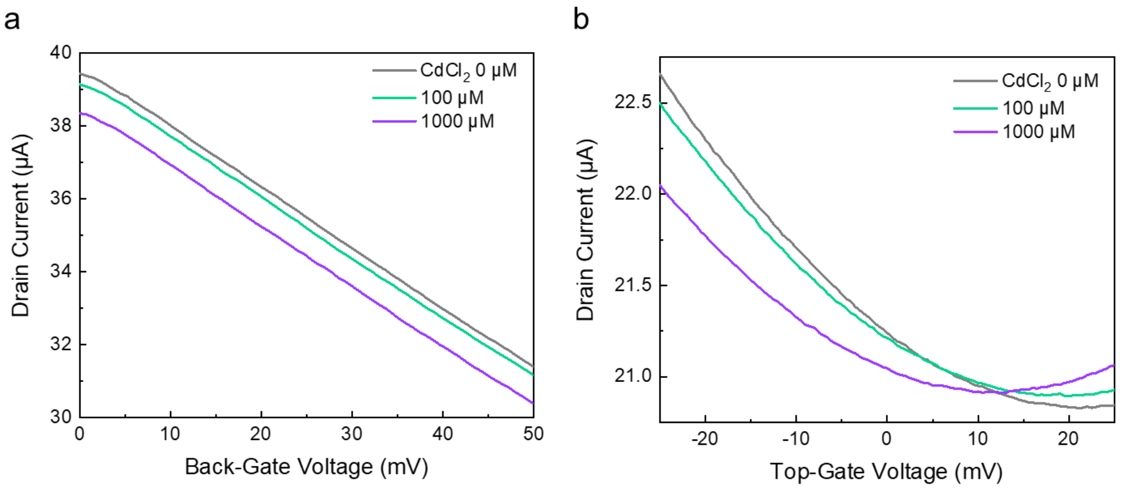

Figure S3. Transfer characteristics when cadmium chloride was introduced into (a) the TTHA-modified and (b) the terpyridine-modified graphene. These transfer characteristics were measured by applying the drain voltage of 50 mV with sweeping the top-gate voltage using a Ag/AgCl reference electrode.[2] The transfer characteristics of the graphene-based sensors were measured using a source-measurement unit (B2912A, Keysight Technologies, USA) at 23 °C.

References

1. Ohno, Y.; Maehashi, K.; Matsumoto, K., Label-Free Biosensors Based on Aptamer-Modified Graphene Field-Effect Transistors. *Journal of the American Chemical Society* **2010,** 132, (51), 18012-18013.
2. Sakamoto, Y.; Ikuta, T.; Maehashi, K., Organic Molecular Detection without Debye-Length Limitation by Desorption of Receptor from the Surface of a Graphene Field-Effect Transistor. *ACS Applied Nano Materials* **2022,** 5, (10), 15642-15650.
